# Development and Preliminary Validation of an Emotional Readiness Tool for End-of-Life Care Volunteers

**DOI:** 10.3390/healthcare14203505

**Published:** 2026-10-18

**Authors:** Zhuyun Lin, Iris K. N. Chan, Vivian Weiqun Lou

**Affiliations:** 1 Faculty of Social Science, The University of Hong Kong, Pokfulam Road, Hong Kong; 2 The Hong Kong Society for Rehabilitation, 1/F, HKSR Lam Tin Complex, 7 Rehab Path, Lam Tin, Kowloon, Hong Kong; 3 Sau Po Centre on Ageing, 11/F, T.T. Tsui Building, The University of Hong Kong, Pokfulam Road, Hong Kong

**Keywords:** end-of-life care, emotional readiness, screening tool, volunteer assessment

## Abstract

**Background:** Volunteers play an important role in community-based end-of-life care (EoLC), yet few structured approaches are available to identify prospective volunteers’ emotional support and preparation needs before service engagement. This study developed and preliminarily evaluated an Emotional Readiness Tool (ERT) as a profile-based screening framework. **Methods:** A cross-sectional study was conducted among 1372 adults who registered for a community-based EoLC volunteer training programme between 2022 and 2024. The 13-indicator ERT covers emotional regulation capacities, death-related experiences, and interpersonal capacities. Exploratory factor analysis (EFA), component-specific internal consistency analysis, correlations with external psychological measures, and exploratory subgroup comparisons were conducted. **Results:** The sample was predominantly female and highly educated. EFA identified coherent clusters among perceived stress and depressive symptom indicators, whereas death-related and interpersonal indicators did not form common latent factors. Because the ERT combines indicators with different response formats and does not currently provide a validated overall score, cutoff, or suitability classification rule, the factor analysis was interpreted as an exploratory examination of association patterns rather than confirmation of a unified latent structure. Emotional regulation capacities were associated with death anxiety and holistic well-being, while demographic differences were generally small. **Conclusions:** The ERT is best understood as a multidimensional, profile-based screening framework rather than a conventional psychometric scale. It may support individualized discussion, training, supervision, and role matching, but should not be used as a diagnostic, gatekeeping, or exclusion tool. Further research should evaluate its temporal stability and predictive and criterion-related utility.

## 1. Background

Volunteers are essential in end-of-life care (EoLC), providing emotional support, companionship, and practical help that benefit patients and families, particularly in community and home-based settings [1,2]. The emotional demands of EoLC have been well documented in recent reviews and observational studies [3,4], emphasizing the importance of assessing volunteers’ emotional readiness before deployment. Higher emotional intensity is associated with maladaptive responses such as rumination [5], suggesting that effective emotion regulation is critical for maintaining psychological stability in this demanding role.

Despite this recognition, screening efforts in EoLC have predominantly focused on patients or paid professionals [6,7], with practical screening frameworks for volunteers, particularly in community-based settings, remaining scarce and rarely designed to capture emotional preparedness. While existing studies examined various aspects of EoLC volunteering, including training design [8,9], recruitment [4], volunteers’ characteristics [10], emotional experiences during service [11], and motivational factors [12,13,14], considerably less attention has been directed toward the conditions that make participation itself sustainable. We address this gap by developing a concise emotional readiness screening framework to identify prospective volunteers’ support needs across three domains: emotional regulation capacities, death-related experiences, and interpersonal capacities.

### Emotional Readiness

The concept of emotional readiness has been examined across several life transitions, including adoption [15], family justice proceedings [16], and mediation [17]. Across these contexts, emotional readiness has been conceptualized in relation to emotion regulation, self-awareness and communicative capacity [17], the emotional states that facilitate constructive decision-making in high-stress relational contexts [16], and stage-based preparedness for adoption [15]. Building on this literature, we conceptualize emotional readiness as a dynamic and malleable state that can be systematically evaluated and supported, rather than as a fixed dispositional trait inherent to the individual.

EoLC volunteering inherently involves engagement with death, dying, loss, and suffering. Individuals who apply to EoLC volunteer programmes are likely to possess at least some awareness of the emotionally demanding natureof the role [3,4]. However, awareness of these emotional demands does not necessarily imply preparedness to manage them; studies of hospice and palliative care volunteers indicate that emotional strain frequently arises during service even among those who entered the role knowingly and with clear motivation [11,18,19]. Key questions remain regarding whether prospective volunteers have sufficient emotional capacities to sustain their involvement [5,20], how previous experiences of loss and caregiving shape their responses to EoLC work [21,22], and whether they can effectively navigate the interpersonal challenges encountered during service [23,24]. A screening framework that captures these multiple dimensions of readiness can help organizations identify volunteers who may benefit from additional support or tailored training before deployment.

Drawing on conceptualizations of emotional readiness as the capacity to engage constructively with emotionally demanding situations while maintaining adaptive functioning [15,16,17], the screening framework encompasses indicators that may influence an individual’s preparedness for EoLC volunteering. Previous theories of emotion regulation have suggested that individuals’ abilities to engage effectively with emotionally challenging situations are influenced by their current emotional functioning, capacity for emotional adjustment, and access to psychological resources [20,25].

Perceived stresses and depressive symptoms included as indicators of current emotional regulation capacities have been widely used to assess individuals’ psychological well-being, coping resources, and adjustment to challenging life circumstances [26,27]. Elevated perceived stresses reflect situations in which individuals perceive environmental demands as exceeding their coping resources [28], while depressive symptoms indicate emotional distress and diminished psychological functioning [27,29]. Both have been consistently associated with reduced well-being and impaired capacity to cope with challenging caregiving situations [23,30,31]. In a screening context, identifying elevated stresses or depressive symptoms among prospective volunteers can indicate the need for enhanced training support or adjusted role expectations during service placement. Thus, emotional regulation capacities were operationalized through perceived stresses and depressive symptoms.

Additionally, previous exposure to death, loss, and caregiving may shape individuals’ perspectives on dying, bereavement, and caregiving, thereby providing contextual information relevant to EoLC volunteering [14,32]. Hence, experiences of bereavement and prior end-of-life caregiving were included as death-related experiences in this framework. Such experiences may increase vulnerability through unresolved grief, emotional burden, or reactivation of loss-related distress [21,33]. At the same time, existing studies suggest that exposure to adversity and caregiving experiences can also facilitate personal growth, meaning-making, and greater preparedness for supporting others facing similar circumstances [11,18]. For screening purposes, these are understood not simply as risk factors, but as contextual indicators that may either deplete or strengthen emotional resources depending on how they have been processed and integrated by the individual.

Beyond emotional regulation capacities and death-related experiences, EoLC volunteering requires the capacity to engage effectively with patients, family members, volunteers, and healthcare professionals. Interpersonal capacities in this context are regarded as two dispositional tendencies that shape how a volunteer manages emotionally charged encounters and that are relevant to identify at the point of registration. The first is openness, understood as the capacity to remain engaged when confronted with values, beliefs, or decisions that differ from one’s own. In EoLC settings, discussions surrounding death, dying, and care preferences are often essential for supporting communication and facilitating person-centred care planning [34], yet such conversations remain constrained by social taboos, avoidance, and divergent beliefs about mortality [34,35]. Volunteers routinely encounter families whose views on disclosure, treatment withdrawal, religious ritual, or the meaning of a “good death” diverge from their own. Individuals who experience substantial discomfort in such situations may withdraw, redirect the conversation, or subtly signal disapproval, each of which can compromise the family’s experience of being accompanied without judgement. Openness is therefore included as an indicator of the disposition to tolerate difference.

The second tendency is overcommitment, understood as the inclination to become so emotionally invested in another person’s situation that one’s own well-being is affected. EoLC volunteering often involves the development of emotionally meaningful relationships with patients and families. While empathic engagement is central to the volunteer role, excessive emotional involvement may place volunteers at risk of emotional burden and exhaustion. Existing studies have highlighted the importance of maintaining appropriate boundaries and managing emotional involvement in relationships with patients and families [23,24]. Thus, overcommitment is applied to measure the interpersonal capacity. However, openness and overcommitment are conceptually distinct and are not assumed to covary: a volunteer may be highly receptive to differing views while maintaining clear emotional boundaries or may struggle with difference while also over-identifying with those they accompany. They are accordingly retained as separate single-item indicators rather than combined into an interpersonal subscale.

Drawing on the conceptualization of emotional readiness as preparedness to engage with the emotional and interpersonal demands of end-of-life care, the ERT is developed as a multidimensional, profile-based screening framework encompassing three domains: emotional regulation capacities, death-related experiences, and interpersonal capacities. Because several indicators contribute distinct contextual information rather than reflecting a shared underlying construct, the ERT is conceptualized as a formative framework rather than as a psychometric scale; hence, no overall total score is calculated. Within this framework, screening refers specifically to identifying support, training, supervision, and role-matching needs at the point of registration; it does not refer to determining suitability for volunteering, diagnosing psychological conditions, or excluding applicants from service, and these uses are explicitly outside the intended scope of the tool. The present study thus examines the empirical structure of the framework, specifies the types of validity evidence currently available, and delineates the boundaries within which the framework may responsibly be applied.

## 2. Methods

### 2.1. Research Design

This study was conducted within the framework of the Jockey Club End-of-Life Community Care Project (JCECC), a community-based end-of-life care initiative launched in Hong Kong in 2016. The project was established through a collaboration between The University of Hong Kong and four non-governmental organizations with the aim of strengthening community-based EoLC services and supporting the preference of terminally ill individuals to remain in community settings whenever possible.

As part of the service model, trained volunteers were integrated into multidisciplinary care teams to provide practical, physical, and psychosocial support to patients and their family members. Individuals interested in becoming EoLC volunteers were required to register prior to attending volunteer training. However, registration for the training programme did not automatically qualify individuals as active volunteers. To be admitted to volunteer service, participants were required to complete the training curriculum and satisfy practicum and service requirements established by the programme.

A cross-sectional design was adopted for the present study. During registration, applicants completed a registration form that collected demographic information, responses to the Emotional Readiness Tool, and information regarding previous volunteering, caregiving, and end-of-life care experiences. In addition, registrants were invited to complete a pre-training questionnaire (T_0_), which assessed volunteer-related competencies and external psychological measures, including death anxiety and holistic well-being. For the purpose of this study, data from the registration form and the pre-training questionnaire were linked and analyzed to evaluate the preliminary psychometric properties of the ERT. Hence, the present study evaluated this tool among prospective volunteers at the initial registration stage.

### 2.2. Participants & Recruitment

Participants were adults aged 18 years or above who had self-selected into the community-based EoLC volunteer training programme. Recruitment was conducted territory-wide across Hong Kong through social service organizations, the project website, social media promotion, and the project’s online volunteer self-learning platform. Participation in the research was voluntary, and no financial or non-financial incentives were offered for completing the registration form or the pre-training questionnaire.

The present study utilized registration data collected between 2022 and 2024. A total of 1544 registration records were initially obtained. To ensure data quality, records were reviewed using names, registration IDs, email addresses, and telephone numbers to identify duplicate registrations. After removing duplicate records and incomplete registrations, a final sample of 1372 participants was retained for analysis of the ERT. Specifically, 250 registered in 2022, 497 in 2023 and 625 in 2024.

Among these participants, 537 (39.1%) completed the pre-training (T_0_) questionnaire and had usable data on the external measures of death anxiety and holistic well-being. This subsample reflects both the exclusion of the 2022 T_0_ data, which were unusable because of an administration error in the measures, and the voluntary nature of the 2023–2024 pre-training questionnaire. Analyses examining preliminary construct-related evidence were conducted using this subsample (*n* = 537), whereas EFA, component-specific reliability analysis, and exploratory subgroup comparisons were conducted using the full registration dataset (*N* = 1372).

### 2.3. Measurements

Death Anxiety: The Chinese version of the 15-item Death Anxiety Scale (CDAS) was used to assess participants’ death anxiety. In its original format, items used a true–false response format, with higher scores indicating higher levels of death anxiety [36]. The Chinese version was translated by Wu et al. [37], who reported acceptable internal consistency (Cronbach’s α = 0.60). In this study, we adopted their translation using a 5-point Likert format from 1 (“Completely disagree”) to 5 (“Completely agree”). Scale scores range between 15 and 75, with higher scores indicating greater death anxiety. In this study, CDAS demonstrated satisfactory internal consistency (Cronbach’s α = 0.797).

Holistic Wellbeing: Holistic well-being was assessed using four single-item self-assessments developed for the present project, in which respondents rated the quality of their health, mood, life, and spiritual well-being. The items were informed by the Body–Mind–Spirit framework in health, which conceptualises well-being as encompassing physical, psychosocial, and spiritual dimensions rather than the absence of illness alone [38]. Each item was rated on a 10-point Likert scale from 1 (“Completely disagree”) to 10 (“Completely agree”), with higher scores indicating better perceived well-being. In this study, the four items showed satisfactory internal consistency (Cronbach’s α = 0.870).

### 2.4. Development of the Emotional Readiness Tool

Drawing on the literature review, an initial pool of 20 items was assembled to assess information relevant to prospective volunteers’ emotional preparation for EoLC service. The initial pool comprised the 14-item Perceived Stress Scale (PSS-14) [26], the two Patient Health Questionnaire items (PHQ-2) [27], and four death-related and interpersonal indicators. Decisions about which items to retain were made by the research team. Items were retained when they (a) addressed one of the three intended domains, (b) could be answered quickly and without clarification at the point of online registration, (c) were directly interpretable by volunteer coordinators without reverse scoring or computation, and (d) were judged acceptable to ask of prospective volunteers before any relationship with the organization had been established.

### 2.5. Item Generation

Emotional Regulation Capacities: The PSS-14, a widely used self-report measure of the degree to which life circumstances are appraised as unpredictable, uncontrollable, and overloading, was reviewed during item development. Following research team discussion, the PSS-14 was adjusted for use. The seven positively worded, reverse-scored items were removed, and the seven negatively worded items were retained. This decision was made to produce a concise screening profile focused on directly interpretable indications of current distress and support needs and to avoid requiring reverse scoring in routine programme use. The retained items directly assessed perceived loss of control, stress, emotional burden, overload, and difficulty coping with demands, and can be interpreted without reverse scoring in routine screening. Responses were rated on a 5-point Likert scale ranging from 0 (“Never”) to 4 (“Always”). Depressive symptoms were assessed using PHQ-2, which evaluated the frequency of depressed mood and loss of interest during the previous two weeks. Items were rated from 0 (“Not at all”) to 3 (“Nearly every day”). The retained PSS-7 items and PHQ-2 items were treated as multi-item components within the emotional regulation capacities domain; reliability estimates were not interpreted as evidence that all 13 ERT indicators form a single scale.

Death-related Experiences: Death-related experiences were assessed using two indicators reflecting personal exposure to death, loss, and end-of-life circumstances. Participants were asked whether they had experienced bereavement of a relative or close friend within the previous two years, recorded as presence or absence. But the number of losses, the time elapsed since bereavement within the two-year window, the cause or circumstances of death, and the subjective emotional impact of these experiences were not recorded. Regarding EoLC experience, we first asked whether the participant was currently caring for a terminally ill person. Participants who answered “Yes” then reported their relationship to that person, which was coded by relational closeness from 0 (“Very distant”) to 4 (“Very close”); participants who answered “No” were coded 0. Thus, the analysis used a single ordinal indicator ranging from 0 to 4, where higher scores indicated the possible higher degree of involvement in caregiving. These indicators aim to capture whether recent death-related exposure occurred. They are accordingly interpreted as contextual information to be explored in conversation during volunteer preparation, rather than as scored evidence of vulnerability or of preparedness.

Interpersonal Capacities: Interpersonal capacities were assessed using two indicators considered particularly relevant to EoLC. Openness, a reversed item, assessed participants’ discomfort with differing opinions and discussing sensitive death-related topics. Responses are scored from 0 to 4, with higher scores indicating greater discomfort and therefore lower openness. Overcommitment assessed the tendency to become excessively emotionally involved in others’ concerns to the extent that personal emotional well-being may be compromised. Responses are scored from 0 to 4. The higher score indicated the higher tendency of overcommitment.

### 2.6. Instrument Review

The ERT was developed through an iterative process involving literature review, discussions among the research team, and consultation with academics and practitioners experienced in end-of-life care and volunteer training. The objective was to develop a concise and practically applicable screening instrument that could be incorporated into volunteer recruitment procedures. Eight social workers experienced in volunteer management reviewed the proposed domains and items to assess their relevance to volunteer screening in EoLC settings. The review process was conducted as an informal expert consultation; reviewers were not asked to systematically rate item relevance, clarity, or representativeness using a structured rating procedure, but they expressed agreement with the items selected. In addition, four experienced volunteers reviewed the tool and provided feedback on its clarity, relevance, and sensitivity.

In practice, based on nearly a decade of experience in volunteer training and ongoing observation of volunteers’ subsequent service engagement, we found that volunteer coordinators and social workers did not use the ERT to rank applicants or make decisions based on a total score. Instead, the tool was primarily used as a structured reference to identify areas that might warrant further discussion, support, supervision, or preparation prior to service involvement. They reviewed individual responses and domain-specific patterns to identify areas that might warrant additional discussion, support, training, supervision, or timing considerations.

Accordingly, indicators were reviewed within their respective domains, including emotional regulation capacities, death-related experiences, and interpersonal capacities, to provide a profile of potential strengths and support needs relevant to EoLC volunteering. Thus, the final instrument comprised 13 items representing three theoretically informed domains relevant to emotional readiness for EoLC volunteering (Table 1). Rather than measuring a single psychological construct, the tool was designed as a brief screening instrument that captures multiple domains considered relevant to preparedness for EoLC service.

### 2.7. Data Analysis

Data was analyzed using SPSS Version 28. Descriptive statistics, including means, standard deviations, frequencies, and percentages, were calculated to summarize participant characteristics and item responses. The ERT incorporated indicators with different response formats, including binary, ordinal, and Likert-scale variables. Before exploratory factor analysis, each indicator was transformed to a z score using *z* = (*x* − mean)/standard deviation so that variables were expressed on a common standardized metric. The mean and standard deviation used in this transformation were those of the indicators in the present sample (*N* = 1372), so that each standardized indicator had a mean of 0 and a standard deviation of 1. This transformation addressed differences in scale range but did not change the categorical or ordinal measurement properties of the indicators.

EFA using Principal Axis Factoring with Promax rotation was conducted to explore patterns among the standardized indicators. Sampling adequacy was evaluated using the Kaiser–Meyer–Olkin statistic and Bartlett’s test of sphericity, and factor retention was informed by eigenvalues greater than one, factor loadings, and conceptual interpretability. Loadings of 0.40 or above were considered substantial. Indicators below this threshold were retained and reported as non-loading because the ERT was designed as a multidimensional framework rather than as a measure of a single latent construct. As polychoric modelling was not employed, the EFA findings should be regarded as preliminary evidence of internal structure rather than as definitive evidence of structural validity.

Internal consistency was assessed only for the multi-item PSS-7 and PHQ-2 components using Cronbach’s alpha. The death-related and interpersonal indicators were conceptualized as standalone contextual indicators rather than reflective scales and were therefore not subjected to internal consistency testing. Preliminary construct-related evidence was examined through exploratory correlations between ERT indicators and external measures of death anxiety and holistic wellbeing, selected on theoretical grounds as constructs potentially relevant to emotional readiness for EoLC.

Exploratory subgroup analyses were conducted to examine variation in individual ERT indicator responses across demographic characteristics, including gender, age, educational attainment, marital status, religious affiliation, and employment status. Group differences were evaluated using one-way analyses of variance (ANOVA), with partial eta squared (η^2^) reported as an indicator of effect size. These analyses were intended to describe demographic variation in ERT responses rather than to establish measurement invariance or provide definitive evidence of validity. Statistical significance was set at *p* < 0.05, and findings were interpreted in conjunction with effect sizes.

To assess the robustness of the findings, a sensitivity analysis was conducted to examine the potential influence of recruitment period and mode of recruitment. The primary analyses were performed using the full sample of participants registered between 2022 and 2024. The EFA was then repeated using only participants recruited through the online volunteer learning platform during 2023–2024. Factor structures obtained from the two samples were compared in terms of the number of extracted factors, factor loadings, and overall interpretability. And the demographic variables across years shared similar distributions.

The study was designed, conducted, and reported in accordance with the Strengthening the Reporting of Observational Studies in Epidemiology (STROBE) statement for cross-sectional studies. While STROBE guided the reporting of the observational components of the study, details related to the development of the screening framework, including indicator generation, item reduction, and construct evaluation, fall outside the scope of STROBE and are therefore reported separately in the relevant subsections.

### 2.8. Statement of Ethics

The study was approved by the Human Research Ethics Committee of The University of Hong Kong (ref. EA220206 on 17 May 2022). All participants provided informed consent electronically during registration and were informed of their right to withdraw at any time. Registration for the volunteer training programme and consent to the research use of registration data were recorded as separate decisions, and consent to research participation was not a condition of admission to training or of subsequent volunteer placement. All questionnaire data were stored on password-protected computers accessible only to authorized members of the research team. Participants were informed of their right to review and request deletion of their questionnaire responses and any audio/video records through the designated project office, in accordance with approved ethical procedures. Because the ERT includes questions concerning depressive symptoms, bereavement, caregiving, and death, responses were accessible only to designated programme staff and were used to inform volunteer preparation and support.

## 3. Results

### 3.1. Participant Characteristics

A total of 1372 individuals registered for the volunteer program between 2022 and 2024. The majority were female (77.8%). Participants were distributed across age groups, with the largest proportion aged 40–49 (23.3%), followed by 30–39 (21.9%) and 18–29 (21.4%). Most participants reported high educational attainment, with 78.6% having completed college or university education or above. Marital status was distributed between single (51.3%) and married/cohabiting (42.1%). Regarding religious affiliation, Christianity was the most common (26.7%), followed by Buddhism (14.3%), while 48.6% reported no religious affiliation. Employment status varied, with 47.4% employed full-time and 11.7% retired. These demographic characteristics indicate that the volunteer pool primarily consisted of highly educated individuals with diverse employment backgrounds and substantial representation of middle-aged adults (Table 2).

### 3.2. Exploratory Factor Analysis

#### 3.2.1. Sampling Adequacy and Suitability

The suitability of the data for factor analysis was first examined. The Kaiser–Meyer–Olkin (KMO) measure of sampling adequacy was 0.868, indicating good adequacy for factor analysis. Bartlett’s test of sphericity was significant, χ^2^ (78) = 4234.628, *p* < 0.001, suggesting sufficient inter-item correlations for factor extraction.

#### 3.2.2. Factor Extraction

Exploratory Factor Analysis was conducted using Principal Axis Factoring with Promax rotation. A factor loading threshold of 0.40 was applied. The analysis included 13 indicators representing emotional regulation capacities, death-related experiences, and interpersonal capacities. Three factors with eigenvalues greater than one were extracted, accounting for 37.384% of the total variance. The resulting factor structure did not correspond to the three theoretically proposed domains, and five indicators did not load substantially on any extracted factor (Table 3).

#### 3.2.3. Pattern of Loadings

As shown in Table 3, the exploratory factor analysis identified three factors. The two depressive symptom items (PHQ-1 and PHQ-2) loaded strongly on a distinct factor (Factor 3), representing depressive affect. Perceived stress items were distributed across two related factors. Factor 1 comprised items reflecting perceived stress associated with uncertainty, loss of control, and emotional tension (PSS1, PSS2, and PSS3), whereas Factor 2 comprised items related to coping burden and feelings of being overwhelmed by life’s demands (PSS8, PSS11, and PSS14). These findings suggest that different aspects of stress may represent distinguishable dimensions of emotional distress among prospective EoLC volunteers. Recent bereavement and current end-of-life caregiving experience did not demonstrate substantial loadings on any extracted factor. Similarly, the interpersonal indicators, namely openness to differing views and overcommitment, as well as PSS12, failed to meet the loading criterion. These findings suggest that death-related experiences and interpersonal characteristics may not function as latent dimensions of the same underlying construct. Rather, they may represent relatively independent contextual experiences and individual characteristics that contribute unique information regarding volunteers’ emotional readiness. Although PSS12 did not meet the statistical criterion for factor retention, it was retained in the tool because of its theoretical relevance and its contribution to preserving the conceptual breadth of the perceived stress domain.

Overall, the EFA results suggest that emotional distress indicators, particularly perceived stresses and depressive symptoms, form coherent clusters within the assessment framework, supporting interpretation of these item sets as reflective subscales, whereas the absence of common factors among several death-related and interpersonal indicators is consistent with their intended use as distinct profile indicators.

### 3.3. Internal Consistency

Internal consistency was evaluated only for the empirically and conceptually coherent multi-item components of the ERT. No overall Cronbach’s alpha was interpreted for the 13-indicator framework because it combined multi-item components with single-item contextual indicators and was not intended to represent a unidimensional latent construct or yield a total score.

Reliability analyses further indicated satisfactory internal consistency for the individual multi-item components. PHQ-2 demonstrated acceptable reliability (Cronbach’s α = 0.771), while the seven perceived stress items showed good internal consistency (Cronbach’s α = 0.787). These findings suggest that the emotional regulation capacities component provides a stable assessment of stress- and depression-related emotional wellbeing among prospective EoLC volunteers. No internal consistency estimate was reported for the death-related or interpersonal indicators.

### 3.4. Construct-Related Evidence

Pearson correlation analyses revealed moderate associations among indicators of emotional regulation capacities and interpersonal functioning. Depressive symptoms were positively correlated with perceived stress (r = 0.435, *p* < 0.001), openness (r = 0.259, *p* < 0.001), and overcommitment (r = 0.340, *p* < 0.001). Perceived stress demonstrated its strongest association with overcommitment (r = 0.563, *p* < 0.001). In contrast, recent bereavement showed no significant correlations with depressive symptoms, perceived stress, openness, and overcommitment. Likewise, current end-of-life caregiving experience demonstrated only weak associations with other indicators.

As shown in Table 4, correlations with external measures further indicated that depressive symptoms, perceived stresses, openness, and overcommitment were associated with higher death anxiety and lower holistic well-being, whereas death-related experience variables showed minimal relationships with these outcomes. These findings suggest that emotional well-being indicators and interpersonal characteristics are more closely related to current emotional regulation capacities, while death-related experiences independently contributed to emotional readiness. These findings support the inclusion of emotional regulation capacities, death-related experiences, and interpersonal capacities as complementary domains within a multidimensional emotional readiness assessment framework.

### 3.5. Exploratory Subgroup Comparisons

Exploratory subgroup comparisons were conducted to describe whether ERT indicators differed across demographic groups (Table 5). Because no directional hypotheses were specified in advance regarding how each demographic characteristic should relate to emotional readiness, these analyses were reported as descriptive comparisons rather than as known-groups validation. Statistically significant differences were observed for several characteristics; however, effect sizes were uniformly small.

Age demonstrated the strongest associations with ERT indicators. Significant age-group differences were found for depressive symptoms (F = 9.63, *p* < 0.001, η^2^ = 0.034), perceived stress (F = 9.40, *p* < 0.001, η^2^ = 0.033), current EoLC experience (F = 4.40, *p* < 0.001, η^2^ = 0.017), openness (F = 3.30, *p* = 0.006, η^2^ = 0.012), and overcommitment (F = 2.96, *p* = 0.012, η^2^ = 0.011). No significant age-group differences were observed for recent bereavement experiences (*p* = 0.637).

For gender, significant differences were observed in recent bereavement experiences (F = 5.04, *p* = 0.025, η^2^ = 0.004), openness (F = 9.49, *p* = 0.002, η^2^ = 0.007), and overcommitment (F = 4.40, *p* = 0.036, η^2^ = 0.003). However, no significant gender differences were identified for depressive symptoms, perceived stress, or current EoLC experience. The associated effect sizes were very small.

Significant differences were also observed across education groups for depressive symptoms (F = 2.88, *p* = 0.022, η^2^ = 0.008) and perceived stress (F = 4.34, *p* = 0.002, η^2^ = 0.013), although effect sizes remained small. No significant differences were found for overcommitment, openness, bereavement experience, or current EoLC experience. No significant differences were found among religion and the indicators.

Differences across marital status groups were generally limited. Significant group differences were found for depressive symptoms (F = 10.05, *p* < 0.001, η^2^ = 0.029), perceived stress (F = 10.33, *p* < 0.001, η^2^ = 0.029), current EoLC experience (F = 2.84, *p* = 0.037, η^2^ = 0.007) and overcommitment (F = 3.31, *p* = 0.010, η^2^ = 0.010). No significant differences were observed for openness or bereavement experiences.

Similarly, employment background was associated with depressive symptoms (F = 2.46, *p* = 0.023, η^2^ = 0.011), perceived stress (F = 3.36, *p* = 0.003, η^2^ = 0.015), current EoLC experience (F = 2.18, *p* = 0.042, η^2^ = 0.010) and openness (F = 2.86, *p* = 0.009, η^2^ = 0.012). No significant differences were identified for overcommitment or bereavement experiences.

Overall, although several demographic comparisons reached statistical significance, all effect sizes were small (η^2^ = 0.003–0.034), indicating that demographic characteristics explained only a limited proportion of variance in the ERT indicators. Statistically significant group differences in this magnitude should not be interpreted as evidence that the ERT validly identifies volunteers who are more or less prepared for EoLC service. They indicate instead that emotional readiness is not strongly determined by demographic characteristics and that individual response profiles, rather than demographic category, should guide volunteer preparation and support.

### 3.6. Sensitivity Analysis

To examine the potential influence of recruitment mode and cohort differences, a sensitivity analysis was conducted by repeating the exploratory factor analysis using only participants recruited through the online volunteer learning platform in 2023–2024 (*n* = 1122). The resulting factor structure was highly comparable to that obtained from the full sample (*n* = 1372). In both analyses, stress-related items formed two distinct factors representing stress reactivity and coping burden, while depressive symptom items formed a separate factor. Openness, overcommitment, and current EoLC experience did not demonstrate substantial factor loadings in either sample. Although recent bereavement emerged as a single-item factor in the restricted sample, the overall interpretation of the ERT remained unchanged.

## 4. Discussion

This study represents one of the first attempts to develop and evaluate a screening instrument specifically designed to assess emotional readiness among prospective EoLC volunteers. Given the emotionally demanding nature of EoLC service [11,19], organizations require practical approaches to identify volunteers who may benefit from additional support, training, or placement in roles at entry point that better match their emotional regulation and interpersonal capacities. The present tool was developed to address this need by assessing emotional regulation capacities, death-related experiences, and interpersonal capacities that may influence volunteering preparation in EoLC settings.

Unlike conventional psychometric scales designed to measure a single latent construct, the emotional readiness tool was developed as a multidimensional profile-based instrument. Consistent with this conceptualization, the EFA did not support the existence of a unified latent structure encompassing all indicators. Instead, perceived stress and depressive symptom items clustered into coherent psychological distress domains, whereas bereavement, end-of-life caregiving experiences, openness to differing views, and overcommitment did not form common factors. These findings suggest that emotional readiness is not adequately represented as a single psychological trait but rather as a profile of emotional, experiential, and interpersonal characteristics that contribute unique information regarding a volunteer’s preparedness for EoLC service.

The findings further provide support for the distinction between emotional regulation capacities and death-related experiences. Perceived stresses and depressive symptoms demonstrated moderate correlations with each other and were consistently associated with external indicators of wellbeing, including death anxiety and holistic well-being. In contrast, bereavement and end-of-life caregiving experiences showed minimal associations with these indicators. This pattern suggests that prior exposure to death and loss does not necessarily translate into current emotional distress. Although bereavement and caregiving experiences may increase vulnerability for some individuals, they may also foster personal growth, meaning-making, resilience, and greater preparedness for supporting others [11,18]. Consequently, these experiences appear to function as contextual indicators rather than direct manifestations of emotional well-being.

The interpersonal indicators also provided meaningful information beyond traditional psychological distress measures. Overcommitment demonstrated moderate-to-strong associations with perceived stresses and depressive symptoms, supporting previous research suggesting that excessive emotional investment and difficulties maintaining boundaries may contribute to emotional burden and burnout among helping professionals and volunteers [19,23]. Individuals who become highly involved in the concerns of others may be particularly susceptible to emotional exhaustion when working with terminally ill patients and their families [19]. This finding reinforces the importance of assessing boundary-management tendencies when preparing volunteers for emotionally demanding roles.

Openness demonstrated significant associations with stress, depressive symptoms, death anxiety, and holistic well-being. This suggests that comfort with engaging in difficult conversations and navigating differing perspectives remains relevant to emotional readiness, even if it operates independently from other emotional indicators. In EoLC contexts, where volunteers frequently encounter diverse beliefs regarding death, dying, spirituality, and treatment preferences, openness may represent an important interpersonal resource that facilitates effective communication and relationship building [39,40].

The exploratory subgroup comparisons offer descriptive context rather than validation evidence. Among the demographic variables examined, age showed the most consistent associations with ERT indicators, particularly depressive symptoms, perceived stresses, interpersonal tendencies, and current caregiving experience. The corresponding effect sizes were nonetheless small. Because the theoretical basis for expecting specific directional differences across demographic groups was not established in advance, the observed group differences are reported as descriptive findings rather than interpreted as evidence of known-groups validity.

Therefore, the results suggest that emotional readiness for EoLC volunteering is best understood as a multidimensional readiness profile rather than a single latent construct. Emotional regulation capacities, prior experiences with death and loss, and interpersonal capacities each contribute unique information regarding a volunteer’s preparedness to engage in emotionally challenging service environments.

## 5. Implications

Given its intended function as a formative readiness framework, the practical value of the ERT should be considered in relation to three connected domains: its implementation within routine organizational processes, the practical interpretability of the response profiles it generates, and its utility in supporting, rather than replacing, professional judgement.

### 5.1. Implementation

At its current stage of development, the ERT is intended for use at the point of registration among prospective volunteers applying for EoLC training. In the present programme, it is embedded in the existing online registration form rather than administered as a separate assessment. This integration is intended to minimize additional administrative burden and to allow potential support needs to be considered before training and service placement are planned. Implementation should extend beyond administering the ERT. Organizations need procedures and resources to explore identified needs through follow-up discussion, tailored training, supervision, debriefing, referral, or appropriate role matching. The ERT should therefore form part of an ongoing support system rather than function as a one-off assessment. Its practical and ethical value depends on whether organizations can respond meaningfully to the needs identified.

### 5.2. Practical Interpretability

Because the ERT is a formative readiness framework rather than a reflective latent-variable instrument, its indicators are not assumed to reflect a single underlying construct. It therefore yields no overall readiness score. Multi-item emotional regulation capacities components may be interpreted at the component level, whereas death-related and interpersonal indicators should be considered individually as contextual information. The absence of a common factor or high internal consistency across all indicators does not undermine the framework’s practical meaning.

Interpretation should focus on the context of each response. Elevated stresses or depressive symptoms may prompt supportive discussion or referral; bereavement and caregiving experience may warrant exploration of their recency, impact, and relevance to volunteering; and overcommitment indicators may prompt discussion of emotional boundaries and support-seeking. No response should be interpreted in isolation, as similar endorsements may carry different meanings for different applicants. Cultural, religious, and family norms should also inform interpretation.

### 5.3. Decision-Support Utility

At this stage, the ERT is a structured readiness-profiling and support-needs framework, not a classification instrument for determining volunteer suitability. It provides no validated cutoff, scoring rule, or decision algorithm. Endorsement of an indicator should prompt further exploration rather than automatic exclusion.

The ERT supports but does not replace professional judgement. Programme staff should interpret responses alongside interviews, motivations, experience, current circumstances, and role demands when considering training, supervision, placement, or role matching. No single response or component score should independently determine eligibility. The ERT is neither a gatekeeping mechanism nor a clinical diagnostic instrument. Bereavement, caregiving experiences, emotional distresses, or boundary-related concerns may indicate support needs but are not grounds for exclusion. Where necessary, staff may recommend further discussion, tailored preparation, enhanced supervision, deferred placement, role adjustment, or professional referral.

As decision-support utility has not yet been established, future prospective studies should examine whether ERT-informed decisions improve preparation, support, placement, and subsequent volunteer outcomes. Until then, the ERT should be used as a structured aid for individualized support and role planning, rather than as a validated measure of suitability.

## 6. Limitations

Several limitations should be acknowledged. First, the ERT was developed through literature review and informal consultation with practitioners and experienced volunteers rather than a formal content-validation process using structured expert ratings and cognitive interviewing. Second, the seven positively worded, reverse-scored PSS-14 items were removed to improve brevity and routine interpretability; although this produced a concise set of directly worded stress indicators, it departed from the full validated PSS-14 and requires further evaluation. Third, the exploratory factor analysis combined binary, ordinal, and Likert-type indicators. Z-score standardization aligned scale ranges but did not address their differing measurement properties, and polychoric or mixed-correlation analysis was not available. The factor pattern should therefore be interpreted as preliminary and descriptive. Fourth, independent split-sample cross-validation and confirmatory factor analysis were not undertaken. A confirmatory model for the full ERT may also be inappropriate unless future research first specifies which components are reflective scales and which are formative or contextual indicators. Fifth, test–retest reliability, predictive validity, and criterion validity were not assessed; the present study cannot establish whether ERT profiles predict training completion, service performance, emotional adjustment, retention, or burnout. Sixth, holistic well-being was assessed using four single-item self-assessments developed for this project rather than a validated instrument. Although these items were informed by the Body–Mind–Spirit framework and showed satisfactory internal consistency, single-item ratings cannot capture the dimensional structure of well-being, and the construct-related evidence involving this measure should be interpreted accordingly. Seventh, participants were self-selected applicants for EoLC volunteer training rather than the general population or active volunteers, limiting generalizability. This point deserves emphasis: those who register for EoLC volunteer training are a distinctive group who have already decided to approach death and dying in a helping capacity, and who may differ from the wider adult population in motivation, prior caregiving exposure, comfort with mortality, and available time and social resources. The observed distributions of stress, depressive symptoms, bereavement, and interpersonal tendencies should therefore not be read as descriptive of adults generally, and the ERT should not be applied outside this context without further evaluation. Additionally, the external-measure analyses were based on a restricted subsample of participants from the 2023 and 2024 cohorts who completed an optional online pre-training survey. Participants who completed these measures may differ systematically from those who did not, and exclusion of the 2022 cohort because of a questionnaire administration error may have introduced cohort effects. The restricted-sample analyses do not establish comparability between participants with and without external-measure data. The observed associations should therefore be interpreted cautiously and replicated in more representative samples with complete external-measure data. Future studies should replicate these findings using independent samples and multiple recruitment cohorts to further establish the robustness and generalizability of the observed relationships. Finally, the study was conducted within a single cultural and service context. Future research should use measurement-level-appropriate analyses, independent validation samples, longitudinal outcomes, and culturally diverse settings.

## 7. Future Research

The most valuable next step would be to follow the present cohort prospectively. Because these participants were assessed at registration, their subsequent trajectories through training, practicum, and service are in principle traceable, and linking baseline ERT profiles to later outcomes would allow the questions this study could not address to be answered directly: whether particular profiles anticipate training completion, practicum performance, sustained volunteering, emotional adjustment during service, or burnout, and equally whether the support offered in response to a profile alters those outcomes. Such a design would permit repeated psychometric assessment over time, establishing the temporal stability of the indicators, and would also show which support needs identified at registration proved consequential in practice and which did not. This is the evidence required before any scoring rule or threshold could responsibly be proposed.

The content of the framework also invites extension. The present indicators capture how a prospective volunteer is currently faring and how they tend to engage with others, but not whether they are willing and able to seek help when difficulties arise. A support-seeking indicator would be a useful addition, since the disposition to approach a coordinator or supervisor when struggling is plausibly protective and is directly actionable in preparation and supervision. More broadly, future versions might incorporate validated measures of meaning in life, emotional self-awareness, and perceived social support, so that the profile represents sustaining resources alongside current burden. Whether such additions improve the framework’s usefulness without compromising the brevity that makes it feasible at registration is itself an empirical question, and one best answered alongside the longitudinal work described above.

## 8. Conclusions

The present study provides preliminary evidence for the ERT as a multidimensional, profile-based screening framework for adults who have registered for EoLC volunteer training. The framework does not measure a single underlying psychological construct and does not generate a validated total score or cut-off. Instead, its multi-item emotional regulation capacities components and death-related and interpersonal indicators offer complementary information that may guide supportive discussion, training adaptation, supervision, and role matching. The ERT should not be used for diagnosis or automatic exclusion from volunteering. Further research should apply measurement-level-appropriate analytic methods, cross-validate the framework in independent samples, and examine its predictive and criterion-related utility for volunteer training, service experiences, retention, and well-being.

## Figures and Tables

**Table 1:** Items Included in the Screening Tool.

No.	Items	Scale	Score
1	(Death-related experience) Have you experienced bereavement of a relative in the past two years?	Binary	0–1
2	(Death-related experience) Current EoLC experience	5-point	0–4
3	(PHQ1) Find everything boring or have no motivation to do anything	4-point	0–3
4	(PHQ2) Low mood, depressed, or hopeless	4-point	0–3
5	(PSS1) How often do you feel uncomfortable about unplanned incidents?	5-point	0–4
6	(PSS2) How often do you feel out of control of important things in your life?	5-point	0–4
7	(PSS3) How often do you feel anxious and stress?	5-point	0–4
8	(PSS8) How often do you find that you cannot take care of everything you have to do?	5-point	0–4
9	(PSS11) How often do you feel angry about things that are outside of your control?	5-point	0–4
10	(PSS12) How often do you think about things that you demand yourself to do?	5-point	0–4
11	(PSS14) How often do you feel overwhelmed by the various difficulties in your life?	5-point	0–4
12	(Overcommitted) How often do you worry about others to a point where your own emotions are troubled?	5-point	0–4
13	(Openness) How uncomfortable were you when you interacted with people with different opinions than yours?	5-point	0–4

Note: PHQ = Patient Health Questionnaire-2 (PHQ-2), a two-item screen for depressed mood and loss of interest; PSS = Perceived Stress Scale, from which the seven negatively worded items of the 14-item version (PSS-14) were retained; item numbering (PSS1–PSS14) followed the original PSS-14 to allow comparison with the source instrument. Death-related experience items and the interpersonal items (Openness, Overcommitted) were developed for this framework and did not derive from an existing scale. Binary = yes/no response; 4-point and 5-point = Likert-type response formats. No total score is computed across the 13 indicators.

**Table 2:** Background Information of the Participants.

Variables		*n*	%
Gender	Male	304	22.2
	Female	1068	77.8
Age	18–29	293	21.4
	30–39	301	21.9
	40–49	319	23.3
	50–59	269	19.6
	60–69	175	12.8
	70–79	15	1.1
Education	Primary school	2	0.1
	Junior secondary school (Form 1 to Form 3)	30	2.2
	Senior secondary school (Form 4 to Form 6)	253	18.4
	College/university or above	1079	78.6
	Others	8	0.6
Marital status	Single	704	51.3
	Married/cohabiting	578	42.1
	Divorced/separated	65	4.7
	Widowed	24	1.7
	Others	1	0.1
Religion	Christianity	366	26.7
	Catholicism	78	5.7
	Buddhism	196	14.3
	Taoism	3	0.2
	Islam	1	0.1
	Chinese traditional beliefs (worshipping gods/Tu Di Gong/ancestors)	51	3.7
	Others	10	0.7
	No religious belief	667	48.6
Employment status	Full time	651	47.4
	Part time	206	15
	Housewife	89	6.5
	Seeking employment/on leave	89	6.5
	Retired	160	11.7
	Unemployed	57	4.2
	Others	120	8.7

**Table 3:** Exploratory Factor Analysis of the Emotional Readiness Tool (*N* = 1372).

Item	Factor 1	Factor 2	Factor 3
	Stress Reactivity	Coping Burden	Depressive Symptoms
PSS1. Feel uncomfortable about unplanned incidents	0.841		
PSS2. Feel out of control of important things in life	0.654		
PSS3. Feel anxious and stressed	0.743		
PSS8. Unable to take care of everything that needs to be done		0.524	
PSS11. Feel angry about things outside your control		0.634	
PSS14. Feel overwhelmed by difficulties in life		0.578	
PHQ1. Little interest or pleasure in doing things			0.764
PHQ2. Feeling down, depressed, or hopeless			0.831
Recent bereavement within past two years			
Current EoLC experience			
Openness to differing views			
Overcommitment			
PSS12. Think about things you demand yourself to do			

Note: *N* = 1372. Principal Axis Factoring with Promax rotation; three factors with eigenvalues greater than one were extracted. Factor 1 = Stress Reactivity; Factor 2 = Coping Burden; Factor 3 = Depressive Symptoms. Blank cells indicate indicators that did not load substantially (<0.40) on any extracted factor, namely PSS12, recent bereavement, current EoLC experience, openness to differing views, and overcommitment. Because the indicators combine binary, ordinal, and Likert-type response formats, the solution is exploratory and descriptive rather than confirmatory.

**Table 4:** Associations of Emotional Readiness Indicators with External Validation Measures.

Indicator	CDAS	Holistic Wellbeing
PHQ-2	0.218 ***	−0.326 ***
PSS-7	0.331 ***	−0.293 ***
Bereavement	−0.042	0.042
Current EoLC Experience	−0.099 *	0.024
Openness	0.222 ***	−0.282 ***
Overcommitment	0.230 ***	−0.182 **

Note: *n* = 537. Values are Pearson correlation coefficients between Emotional Readiness Tool indicators and the external measures. PHQ-2 = two-item Patient Health Questionnaire (depressive symptoms); PSS-7 = seven retained perceived stress indicators; Bereavement = bereavement of a relative or close friend within the past two years; EoLC Experience = current end-of-life caregiving experience; CDAS = Chinese Death Anxiety Scale. * *p* < 0.05, ** *p* < 0.01, *** *p* < 0.001.

**Table 5:** Exploratory Subgroup Comparisons of Emotional Readiness Indicators (*N* = 1372).

Indicator	Age	Gender	Education	Religion	Marital Status	Employment
PHQ-2	9.63 *** (0.034)	NS	2.88 * (0.008)	NS	10.05 *** (0.029)	2.46 * (0.011)
PSS-7	9.40 *** (0.033)	NS	4.34 ** (0.013)	NS	10.33 *** (0.029)	3.36 ** (0.015)
Bereavement	NS	5.04 * (0.004)	NS	NS	NS	NS
Current EoLC experience	4.40 *** (0.017)	NS	NS	NS	2.84 * (0.007)	2.18 * (0.010)
Openness	3.30 ** (0.012)	9.49 ** (0.007)	NS	NS	NS	2.86 ** (0.012)
Overcommitment	2.96 * (0.011)	4.40 * (0.003)	NS	NS	3.31 * (0.010)	NS

Note: *N* = 1372. Cells report the one-way ANOVA *F* statistic with the effect size (η^2^) in parentheses. PHQ-2 = two-item Patient Health Questionnaire (depressive symptoms); PSS-7 = seven retained perceived stress indicators. NS = not significant (*p* ≥ 0.05). * *p* < 0.05, ** *p* < 0.01, *** *p* < 0.001. Effect sizes were uniformly small (η^2^ = 0.003–0.034), indicating that demographic characteristics accounted for only a limited proportion of variance. These comparisons are exploratory and are not presented as evidence of screening validity.

## Data Availability

The data presented in this study are available on request from the corresponding author. The data are not publicly available due to ethical restrictions protecting participant and community confidentiality.
